# Editorial: The 4th NACAN summit proceedings: nutrition and food for a healthy life

**DOI:** 10.3389/fnut.2023.1200954

**Published:** 2023-05-04

**Authors:** Zhi Chai, Lei Hao, Sheau C. Chai, Guoxun Chen, Fanbin Kong, Chaodong Wu, Zhiping Yu, Ling Zhao, Jin-Rong Zhou

**Affiliations:** ^1^Department of Genetics and Genomic Sciences, Icahn School of Medicine at Mount Sinai, New York, NY, United States; ^2^Department of Nursing and Allied Health Professions, Indiana University of Pennsylvania, Indiana, PA, United States; ^3^Department of Behavioral Health and Nutrition, University of Delaware, Newark, DE, United States; ^4^College of Biomedicine and Health, Huazhong Agricultural University, Wuhan, Hubei, China; ^5^Department of Food Science and Technology, University of Georgia, Athens, GA, United States; ^6^Department of Nutrition, Texas A&M University, College Station, TX, United States; ^7^Department of Nutrition and Dietetics, University of North Florida, Jacksonville, FL, United States; ^8^Department of Nutrition, The University of Tennessee, Knoxville, TN, United States; ^9^Nutrition/Metabolism Laboratory, Department of Surgery, Beth Israel-Deaconess Medical Center, Harvard Medical School, Boston, MA, United States

**Keywords:** diet, nutraceutical, metabolism, dietetics, disease, food, nutrition, health

Established in 2012, the North America Chinese Association for Nutrition (NACAN) celebrated its 10th anniversary in 2022 (https://www.nacan-us.org/). As a public, non-profit charitable organization under the US Internal Revenue Code Section 501(c)3 and a Commission on Dietetic Registration (CDR) accredited continuing professional education (CPE) provider to the US registered dietitians, NACAN continues to advance science and education among professionals with Chinese heritage in nutrition, health, and related fields of interest. NACAN aims to promote scientific exchange and networking, facilitate advanced education and training, support dissemination and application of nutrition knowledge, and advocate nutrition-related research and evidence-based practice to promote health and wellness.

Joined by their collaborators and co-sponsors, the Chinese American Food Society (CAFS), China Food Publishing Co (CFPC), Guangdong Nutrition Society (GNS), Hebei Nutrition Society (HNS), and College of Food Science and Engineering at Nanjing University of Finance & Economics (NUFE), NACAN hosted the 4th NACAN *Frontiers in Nutrition* Summit—The Joint Nutrition Scientific Symposia with meeting theme—“Nutrition for a healthy life: from the production of foods to healthy eating for everyone” in July of 2022. In collaboration with the journal *Frontiers in Nutrition*, NACAN launched a Research Topic to showcase the research contributed to the summit and beyond. This Research Topic collected research papers to address the following four themes:

- Emerging fields of research: New and innovative areas, especially revolutionary and/or multi-disciplinary approaches that address fundamental questions in nutrition, food, and health sciences.

- Scientific base of nutrition: Mechanistic studies that decipher the underlying links between nutrients, diet, and chronic diseases.
- Agriculture and food production: Functional foods, bioactive compounds, nutraceuticals, and novel food processing technologies that improve human health.
- Nutrition and public health: Clinical and population-based studies that examine the role of diet, food, and nutrition in health and disease.

Nutrition intervention is important for disease management. Li Z. et al. reviewed the potential mechanisms of diabetes mellitus combined with Alzheimer's disease and suggested nutrition therapy to reduce the risk of the comorbidity of those two diseases, which include the management of dietary intake, dietary patterns (e.g., ketogenic diet, Mediterranean diet), and nutrition supplements (e.g., probiotics, vitamins, minerals, omega-3 fatty acids).

Opportunities and challenges co-exist for nutritional intervention in childhood cancers. Wang K. et al. discussed the nutrition status among pediatric cancer patients. They reported nutrient dependencies on amino acids, glycolysis and oxidative phosphorylation, lipids, vitamins, and minerals. Several dietary modifications were reviewed, including calorie restriction, ketogenic diet, nutrient restriction, and nutrient supplementation.

Type 2 diabetes mellitus (T2DM) is the most common type of diabetes that has high comorbidity with obesity, renal impairments, and vitamin D (VD) deficiency. Multiple nutrients have been found to alleviate T2DM, shown in rodent models and in human subjects. Using Zucker diabetic fatty rats that were fed VD deficient diet, Wang D. et al. demonstrated 1,25(OH)_2_D_3_ improved urinary Cu, Zn, Se, and Mo excretions, suggesting a protective effect against diabetic renal impairment. Using *in vitro* and *in vivo* models in another study, Wang Y. et al. reported that an olive-derived elenolic acid stimulated GLP-1 and PYY secretion, along with a series of ameliorations on the perturbated metabolic variables in obese diabetic mice. In a meta-analysis, Zhang, Ding et al. concluded that biotin (vitamin B7) supplementation may reduce fasting blood glucose, total cholesterol, and triglyceride levels.

Obesity is a major public health concern, which is closely related to chronic inflammation and brown adipose tissue dysfunction. Bae et al. examined the effects of naringenin, a citrus flavanone, on adipocyte browning, thermogenic activation and brown adipogenesis *in vitro*. The results suggest that naringenin may promote the development of functional brown adipose tissue, in part through PPARγ activation.

Cognition decline is associated with old age and chronic diseases, including neurodegenerative or neuropsychiatric disorders, diabetes and chronic kidney disease. Using an adenine-induced cognitive impairment mouse model, Abdolmaleky et al. demonstrated that mice treated with oligo-lactic acid (LAP) and fermented soy extract (IMB) had significantly improved cognitive performance. The neuroprotective effects of LAP and IMB are mediated by favorable alteration in the gut microbiome as well as through their anti-neuroinflammatory properties.

Inflammation contributes to the development of non-alcoholic fatty liver disease (NAFLD) and non-alcoholic steatohepatitis (NASH). While NAFLD is generally considered a benign condition, NASH can further progress to cirrhosis, liver failure, and liver cancer. Li H. et al. reviewed how hepatic and extra-hepatic signals activate the stimulator of interferon genes (STING) in macrophages, which triggers hepatic inflammation, a key factor driving the development of NAFLD/NASH. While more research is needed, current evidence suggests that STING plays a role in promoting liver inflammation and damage. Targeting the STING pathway may therefore represent a potential therapeutic approach for managing NAFLD/NASH in the future.

Vitamin A (VA) is vital for an individual's general health. Zhang, Tian et al. found that VA status regulates body weight, glucose, lipid levels, and hepatic gene expression in Zucker lean and Zucker fatty male rats. Furthermore, replenishing VA through the diet can restore the expression levels of hepatic genes for glucose and lipid metabolism in Zucker lean male rats.

Sphingomyelin and its metabolites have diverse biological functions. Yang and Chen explored their distribution, digestion, absorption, and metabolic pathways, and reviewed their nutritional functions in chronic metabolic diseases. While endogenous sphingomyelin production is linked to pathological changes, dietary supplementation has been shown to maintain lipid homeostasis. The paper also evaluated their possible implications in modern food preparations, skin improvement, delivery systems, and oil organogels.

In summary, the Joint Nutrition Scientific Symposia and all papers collected in the Research Topic offer insights into the development of research in nutrition and food for a healthy life.

## Author contributions

All authors listed have made a substantial, direct, and intellectual contribution to the work and approved it for publication.

